# Landscape of Epstein-Barr virus gene expression and perturbations in cancer

**DOI:** 10.21203/rs.3.rs-3911441/v1

**Published:** 2024-02-02

**Authors:** Ka-Wei Tang, Yarong Tian, Guojiang Xie, Alan Bäckerholm, Isak Holmqvist, Diana Vracar, Jianqiong Lin, Jonas Carlsten, Sanna Abrahamsson, Zhentao Liu, Yufei Huang, Kathy Shair

## Abstract

Epstein-Barr virus (EBV) is the causative agent for multiple neoplastic diseases of epithelial and lymphocytic origin1-3. The heterogeneity of the viral elements expressed and the mechanisms by which these coding and non-coding genes maintain cancer cell properties in vivo remain elusive4,5. Here we conducted a multi-modal transcriptomic analysis of EBV-associated neoplasms and identified that the ubiquitously expressed RPMS1 non-coding RNAs support cancer cell properties by disruption of the interferon response. Our map of EBV expression shows a variable, but pervasive expression of BNLF2 discerned from the overlapping LMP1 RNA in bulk sequencing data. Using long-read single-molecule sequencing, we identified three new viral elements within the RPMS1 gene. Furthermore, single-cell sequencing datasets allowed for the separation of cancer cells and healthy cells from the same tissue biopsy and the characterization of a microenvironment containing interferon gamma excreted by EBV-stimulated T-lymphocytes. In comparison with healthy epithelium, EBV-transformed cancer cells exhibited increased proliferation and inhibited immune response induced by the RPMS1-encoded microRNAs. Our atlas of EBV expression shows that the EBV-transformed cancer cells express high levels of non-coding RNAs originating from RPMS1 and that the oncogenic properties are maintained by RPMS1 microRNAs. Through bioinformatic disentanglement of single cells from cancer tissues we identified a positive feedback loop where EBV-activated immune cells stimulate cancer cells to proliferate, which in turn undergo viral reactivation and trigger an immune response.

### Main

Epstein-Barr virus (EBV) is estimated to cause 120,000-357,900 cases of neoplasms worldwide each year and 1.8% of all cancer deaths are attributed to EBV-associated malignancies[1,2].

Originally identified in the neoplastic cells of endemic Burkitt's lymphoma (eBL), EBV is the first discovered human tumor virus and infection is extremely common with more than 95% seropositivity among adults worldwide[3,6]. EBV was subsequently implicated as the causative agent in other hematological neoplasms including a fraction of sporadic Burkitt's lymphoma (sBL). The vast majority of nasopharyngeal carcinoma (NPC) and approximately every tenth gastric adenocarcinomas (GAC) are associated with EBV infection and these epithelial malignancies constitute more than 80% of all known EBV-associated cancer cases[7]. Cell lines have been derived from primary tumors or by immortalization of primary B-cells by EBV (lymphoblastoid cell lines)[8-10].

In recent years bulk transcriptome (RNA-Seq) and single-cell sequencing data (scRNA-Seq) from virus-associated neoplasms have become publicly available[11-16]. Viral gene expression in neoplasms have been shown to be associated with the respective known viral oncogenes, for example E6 and E7 in human papillomavirus associated cancer and T-antigen in polyomavirus associated cancer[17]. Multiple RNA-Seq studies have shown that EBV mRNA predominantly originate from the BamHI-A/I region, in which none of the known EBV-oncogenes are encoded. In a single gastric adenocarcinoma, it was initially suggested that the rightward transcribed long non-coding RNA *RPMS1* was expressed in the tissues[5,17]. However, subsequent analysis of larger cohorts of EBV-expressing neoplasms suggested that the transcripts were encoded by the overlapping leftward transcribed genes[4,18]. EBV genomic fragments containing these regions have been shown to have transformative properties[19,20].

*EBV in primary tissues and cell lines*

We started with bulk transcriptome data from publicly available datasets originating from four malignancies with known EBV-association, NPC, GAC, eBL and sBL, as well as EBV-associated tumor-derived cell lines (ECL) and EBV-transformed lymphoblastoid cell lines (LCL)[10,21-24]. The datasets were mapped against the EBV reference genome (Figure 1a, Extended Data Table 1). The EBV fraction was calculated as parts per million (ppm) and datasets with lower than 10 ppm EBV reads were classified as EBV-negative and were not processed further[25]. In accordance with known epidemiology, the majority of NPC and eBL tumors contained more than 10 ppm EBV reads and conversely, the majority of GAC and sBL did not contain any EBV reads (Figure 1b)[2]. The EBV fraction in NPC varied between 14-1,131 ppm, in GAC 15-331 ppm, in eBL 18-470 ppm, and in sBL 149-502 ppm, likely reflecting the purity of tumor cell in the biopsy (Extended Data Fig. 1a). Of the fourteen ECL, eight cell lines contained less than two ppm EBV reads, while the remaining six contained 135-286 ppm EBV reads. All LCL datasets contained, as expected, high levels of EBV reads ranging from 816-16,946 ppm. The EBV gene expression of 156 tumors with minimum 10 ppm EBV-reads originating from 106 NPC, 30 GAC, 16 eBL, and 4 sBL were further processed. The detected EBV-reads were then aligned to the viral genome on coverage plots (Figure 1c) (Supplementary Information; EBV RNA). Of the average EBV coverage in these primary tumors, RNA from the adjacent BamHI-A and I region constituted 88% (standard deviation ±15%) in NPC, 92% (±6%) in GAC, 85% (±19%) in eBL, and 92% (±7%) in sBL (Extended Data Fig. 1b). In contrast, with the exception of the ECL C666-1 (90% BamHI-A/I RNA), on average 6% (standard deviation ±7% and ±4% respectively) of the EBV RNA, in the ECL and LCL datasets, aligned to the BamHI-A/I region.

*EBV genes expressed in cancer*

The majority of the EBV RNA mapped to the BamHI-A/I regions in primary tumors aligned to areas in which multiple genes overlap (Figure 1d). In order to discern the RNA elements within the BamHI-A/I regions we conducted RNA peak (Extended Data Fig. 2a,b), strand-specificity (Figure 1d third panel above/below baseline) (Supplementary Information; EBV RNA), transcription start site (Figure 1e) (Supplementary Information; *RPMS1* transcription start site), RNA splicing (Figure 1f) (Supplementary Information; BamHI-A/I splice-junctions), and polyadenylation signal analyses (Figure 1g) (Extended Data Table 2). The results all supported that the major transcript in the four EBV-positive tumor types originated from the long non-coding RNA *RPMS1*. However, RNA located in the *RPMS1* introns not coupled to the constitutive exons (Figure 1d, *BALF5* mid-region) suggests the presence of other RNA elements. Co-transcriptional activation of these elements was observed in an inducible *RPMS1* promoter mutant Burkitt's lymphoma cell line (Supplementary Information; EBV RNA, BamHI-A/I splice-junctions and Namalwa modified cell lines). An adapted full-length single-molecule sequencing of *RPMS1* (Extended Data Fig. 2c-e)[26], allowed us to identify three new rightward elements which we named BamHI-A rightward elements, *BARE1-3*, (Supplementary Information; BamHI-A rightward elements). We amended the EBV reference genome (NC_007605.1) with the new annotations for BAREs and aligned the EBV reads to the new genome. Non-aligned EBV reads were plotted and additional gene segments were added to increase the aligned fraction producing an >95% EBV mappability for all except three datasets (Extended Data Table 1). In order to produce an unbiased quantification of the global EBV expression in the different tumor types and cell lines, we applied the length-adjusted tpm-values of house-keeping genes and EBV genes by normalizing them to the content of the entire dataset (Extended Data Table 3). Calculation of tpm-values for EBV genes required additional modifications due to overlapping regions exemplified by *RPMS1* and *BAREs*, for which we calculated the tpm-values relative to their unique regions and the overlapping regions as a fused gene, *RPMS1/BAREs* (Figure 2a-f) (Extended Data Table 4). The tpm-values of *RPMS1* were thus calculated relative to its first four exons and *BAREs* relative to respective first exons, while the fused *RPMS1/BAREs* was calculated based on *RPMS1* exons V-VII which all rightward BamHI-A/I genes share. This division creates a bias with artificially lower values for the *RPMS1* and *BAREs* unique regions, due to 5'-degradation of mRNA which is overrepresented in polyA-enriched libraries (Figure 2g). The similar division was also employed for the *LMP1/BNLF2* and *LMP2A/B* genes (Figure 2h-i).

Although no absolute threshold can be set, we chose to mainly consider genes with tpm-values over 5 (Supplementary Information; Tpm-threshold) (Extended Data Fig. 3a-c). The normalized tpm-values of EBV genes in tumors are diluted due to the inclusion of stromal cells[27]. Conversely, using a low tpm-cutoff will include viral genes that are likely to originate from few cells undergoing reactivation, which are responsible for the high viral background. A high degree of EBV reactivation can be observed in three tumors, NPC3, eBL1 and eBL5, in which global viral transcription including oriLyt RNA (eBL1) can be observed (Extended Data Fig. 4) (Supplementary Information; EBV gene expression). With the exception of tumors with EBV reactivation, high expression of viral genes was only observed for the genes *RPMS1/BAREs* (77% of tumors), *LMP1/BNLF2* (10% of tumors) and *LMP2A/B* (1% of tumors). Intermediate expression of *RPMS1/BAREs*, *LMP1/BNLF2* and *LMP2A/B* were detected at 15%, 14% and 8% of tumors respectively. Low expression of *RPMS1/BAREs* were detected in the remaining tumors (8%) and *LMP1/BNLF2* and *LMP2A/B* were observed in 22% and 31% of tumors. Thus, *RPMS1/BAREs* were expressed in all tumors, on average 77 tpm. The most abundant and common protein coding RNA originated from the *LMP1/BNLF2* gene. *LMP1* has a 2 kb unique 5'-region separated from *BNLF2*, compared with 840 base pairs for

*RPMS1* and *BARE1*, and therefore less likely to be false negative due to RNA degradation. Amongst the NPC, 61 datasets had >5 tpm *LMP1/BNLF2*, but only 29 datasets had *LMP1* expression >5 tpm (Figure 2h). The majority (46/61) NPC had at least two-fold higher tpm-value of *LMP1/BNLF2* compared with only *LMP1*. This indicates that the RNA originated from *BNLF2* and not *LMP1* in the majority of neoplasms[28,29]. In contrast, *BNRF1* which is located within the last intron of *LMP2A/B* and shares 448 base pairs 3'-UTR with *LMP2A/B* was not expressed in the neoplasms (Figure 2i). In contrast, in all six EBV-expressing cell lines (ECL), *BHRF1* can be detected at low or moderate levels in the lymphoma cell lines. Multiple *EBNAs* were expressed, as well as *LMPs*. However, compared with primary neoplasms *RPMS1/BAREs* were only expressed at low levels in two lymphoma ECL and at high levels in the NPC cell line C666-1 (Figure 2e). In LCL the EBV expression encompassed almost the entire viral genome in all datasets, which likely reflect the different cell/virus cycle stages in the *in vitro* culture and a distinct proliferative drive compared with the tumor samples (Figure 2f).

Comparison between EBV-positive and their EBV-negative counterparts has previously described that EBV-tissues have an enrichment of genes correlating to an upregulation of proliferative and immune signaling pathways[4,22,30]. To identify EBV-induced perturbations we applied a variance stabilizing transformation normalization using all tumors in each respective cancer category to find the largest differences between the tumors irrespective of EBV-status[31] (Extended Data Fig. 5a). The principal components with largest Euclidean distance between the midpoint of the EBV-positive and EBV-negative datasets showed the genes that most likely influenced by the presence of EBV (Fig. 2j, Extended Data Fig. 5b-e). A pathway enrichment analysis for each of the principal components showed that all the EBV associated cancer types contained perturbations of *MYC* and *E2F* targets, G2M cell cycle progression and interferon response. However, using bulk RNA sequencing, perturbations may arise from interindividual differences and stromal cell composition. Also, cell pathways activated/down-regulated in both

EBV-positive and EBV-negative tumors would not be detected. We therefore extended our analyses to scRNA-Seq datasets, which allows for the identification of perturbations in specific cell populations.

*EBV expression in scRNA-Seq NPC samples*

We processed scRNA-Seq data of 532,122 cells originating from four NPC studies consisting of 63 primary nasopharyngeal samples including 52 NPC and 11 non-tumor (NT) tissues (Figure 3a, Extended Data Table 1)[11-13,16]. Amongst the NPC datasets, the single cell preparation in Study 1, 3 and 4 (scNPC1-15 & 32-52, scNT1 & 9-11) was achieved by direct dissociation of primary tissue. In Study 2 (scNPC16-31 and scNT2-8), the epithelial cells were first enriched by flow cytometry and then remixed with stromal cells. Cell type specific clusters of the nasopharyngeal tissue showed that epithelial cell content in the tumor varied among the samples in the different studies, 0.7-37.3% in Study 1, 0.3-18.5% in Study 3 and 0.0-7.5% in Study 4 (Extended Data Table 5). In Study 2 (0.2-69.2%) the epithelial cells were enriched and the results were therefore not representative of an unperturbed tissue. The variation of epithelial cell content likely reflects the biological differences, but also the efficiency of epithelial cell dissociation. The distribution of cell composition showed that T and B-lymphocytes were the most abundant stromal cell types in both NPC and non-tumor tissues (Extended Data Fig. 6).

EBV reads were detected in the stromal and epithelial cells (Figure 3a). However, a high variation of the fraction of infected cells was observed between patients. No EBV was detected in the non-cancerous samples (scNT), with the exception of scNT5 and scNT11 where one EBV-positive B-lymphocyte was found in each dataset. EBV status for the NPC tissues was clinicopathological analyzed by EBV encoded RNA *in situ* hybridization (EBER-ISH) (Study 1-3) or using an EBV specific antibody (Study 4) (Figure 3b). When comparing the results of scRNA-Seq with the experimental assays, four samples in each group had a discordant EBV status. EBER-ISH had the highest sensitivity for the detection of EBV, and the inability of scRNA-Seq to detect EBV RNA in three tumors could be due to the limited number of input cells, low levels of EBV polyadenylated gene expression and/or the low capture rate of the scRNA-Seq technique[32]. In contrast, EBV was detected in four scRNA-Seq datasets which were negative in EBER-ISH or EBV antibody staining (Figure 3b, marked in grey). The proportion of EBV RNA positive epithelial cells in the EBV positive tumors was highly variable, ranging from 0.4-98.3%.

When we considered the EBV expression in 18 samples that contained more than 100 EBV positive epithelial cells (Figure 3c), *RPMS1/BAREs* was detected in every tumor at high proportions (>46% of EBV-positive cells). Considering the capture rate of the methodology this implies that all cells expressed *RPMS1/BAREs*. *LMP1/BNLF2* and *LMP2/BNRF1* were also detected in every tumor, but at highly variable proportions. The variability was most prominent in *LMP1/BNLF2* ranging from 3.4% to 99%. Even though scRNA-Seq has a lower sensitivity in terms of RNA capture compared to bulk sequencing (Extended Data Fig. 7), the absence of viral RNA background originating from reactivated cells observed in bulk sequencing allows for every viral transcript in scRNA-Seq to be considered. A few reactivated cells as defined by expression of the immediate-early genes *BZLF1/BRLF1* was detected in half of the tumors (Supplementary Information; EBV-positive cells)[33]. Low levels of *EBNA1/3B/3C* can be observed in the majority of tumors. This supports that all NPC expresses *RPMS1/BAREs* at high levels, *LMP1/BNLF2* and *LMP2/BNRF1* at variable levels and possibly *EBNA1/3B/3C* at low levels (Extended Data Table 6). The EBV expression in stromal cells mostly mirrored the expression in epithelial cells, but specific tumors displayed a high degree of reactivation in stromal cells.

The classification of cancer cell status solely based on EBV RNA has its limitations. A high fraction of EBV false negative cells is expected considering the low capture rate of the technology. A proportion of EBV false positive cells caused by indiscriminate uptake of apoptotic bodies from cancer cells by healthy cells would also reduce the correct classification[34]. Analysis by inference of copy-number variants, a pseudo-marker for chromosomal aberrations, allows for cancer cells assignment based on transcription from entire segments of chromosomes instead of a few EBV reads (Figure 3d) (Supplementary Information; Cancer cell identification). With minor exceptions, the pattern of malignant cells within the same tumor displayed a high homogeneity reflecting the clonality of the cancer cells. As expected, the assignment of cancer cells overlapped with the EBV-positive cells, but with an increased sensitivity (Figure 3e-g).

*Host perturbations in cancer cells*

Comparison of cancer cells with healthy epithelial cells from the same tumor removes interindividual bias and the shared microenvironment allows for detection of perturbations in tissue-specific conditions. Furthermore, removal of stromal cells increases the signal of cancer cell specific perturbations. A gene set enrichment analysis based on comparison of equal number of cancer and healthy cells from 42 tumors shows a distinctive pattern shared amongst the EBV-positive tumors (Figure 4a, Extended Data Fig. 8)[35]. EBV-positive cancer cells from the four studies shared eight upregulated pathways compared with corresponding healthy cells for each tumor. Upregulation of five proliferative pathways were observed in the EBV-positive tumor cells as well as basal cells compared with corresponding differentiated cells in seven non-tumor biopsies (Supplementary Information; Epithelial cell classification). Cancer cells from the EBV-negative tumors displayed significantly upregulation of the pro-proliferative mitotic spindle pathway. In the EBV-negative HK1-cells transfected with *RPMS1* miR-BARTs, but not *RPMS1* long non-coding RNA (data not shown), the upregulation of four of the proliferative pathways were reconstituted.

The EBV-positive cancer cells displayed downregulation of immune response[36]; a result not observed in the basal cells, demonstrating that the perturbation observed in the cancer cells is not due to differences in epithelial cell types. Downregulation of interferon response was further confirmed in both HK1-cells transfected with *RPMS1* miR-BARTs as well as in a Burkitt's lymphoma cell line, Namalwa with an induced *RPMS1* promoter, but not in cells expressing ectopic *RPMS1* long non-coding RNA (Extended Data Fig. 9) (Supplementary Information; Namalwa modified cell lines). These findings indicate that the *RPMS1* miR-BARTs induces pro-proliferative pathways and inhibits immune response in cancer cells[37].

In order to identify EBV-induced changes we sorted the genes, based on the number of tumors in which they are perturbed in the same direction, and identified significantly enriched ones (Figure 4b, Extended Data Table 7). The genes were most strongly correlated with down-regulation of oxidative phosphorylation in the EBV-positive cancer cells, likely to be due to the Warburg effect[38]. A strong correlation was also observed with the downregulation of interferon response. The genes involved in immune response downregulation were compiled in order to discern the various pathways (Figure 4c). More than two-thirds of these genes, including MHC1[39], were also shown to be downregulated in HK1 cells transfected with miR-BARTs and Namalwa with an induced *RPMS1* promoter (Extended Data Table 7). The largest proportion of immune genes were regulated by cytokine response. Thus, we analyzed bulk sequencing data of NPCs to detect the gamut of expressed cytokines[40]. The origin of the expressed cytokines was then determined in the scRNA-Seq datasets. The epithelial cells expressed the majority of cytokines of which a few are known to be induced by interferon stimulation (Extended Data Fig. 10a). Amongst the two cell types found in all tumors, B- and T-cells, B-cells produced few cytokines at low levels in most studies. The costimulatory cytokine CD70 was expressed at highest levels in B-cells. Throughout the four studies, the chemokine CCL4 was expressed in a large fraction of T-cells and interferon gamma was expressed at high levels in a smaller subset of cells. The scRNA-Seq data shows that healthy epithelial cells and lymphocytes expresses interferon stimulated genes. Considering that these cells, especially the lymphocytes, constitutes a large proportion of the tumor, this would explain the interferon response upregulation found in the bulk sequencing results (Figure 2j-m). Conversely, the cancer cells exhibit a dysregulated response to interferon. To determine whether this contrasting response we looked at downstream effector genes of interferon gamma (Extended Data Fig. 10b).

No unified difference can be observed in the expression of interferon gamma receptor and downstream kinases between the cancer cells and healthy cells. Multiple genes were expressed at too low levels to allow for a proper comparison. However, the highest expressed *STAT3*-gene were upregulated in cancer cells from all four studies (significantly in three), but not in the EBV-negative tumors (Extended Data Fig. 10b). A weaker trend for *STAT1* downregulation can also be observed. Both *STAT1* and *STAT3* are known to be bound by miR-BARTs[37,41]. The downregulation of interferon and p53 pathways as well as upregulated proliferative pathways are known hallmarks of *STAT*-dysregulation[42]. We therefore propose that the ubiquitously expressed EBV *RPMS1* gene induces interferon response dysregulation through viral microRNA perturbations of *STAT*-expression (Figure 4d).

## References

1 Zapatka, M. *et al.* The landscape of viral associations in human cancers. *Nat Genet* **52**, 320-330 (2020). https://doi.org:10.1038/s41588-019-0558-9

2 Wong, Y., Meehan, M. T., Burrows, S. R., Doolan, D. L. & Miles, J. J. Estimating the global burden of Epstein-Barr virus-related cancers. *J Cancer Res Clin Oncol* **148**, 31-46 (2022). https://doi.org:10.1007/s00432-021-03824-y

3 Farrell, P. J. Epstein-Barr Virus and Cancer. *Annu Rev Pathol* **14**, 29-53 (2019). https://doi.org:10.1146/annurev-pathmechdis-012418-013023

4 Chakravorty, S. *et al.* Integrated Pan-Cancer Map of EBV-Associated Neoplasms Reveals Functional Host-Virus Interactions. *Cancer Res* **79**, 6010-6023 (2019). https://doi.org:10.1158/0008-5472.CAN-19-0615

5 Strong, M. J. *et al.* Differences in gastric carcinoma microenvironment stratify according to EBV infection intensity: implications for possible immune adjuvant therapy. *PLoS Pathog* **9**, e1003341 (2013). https://doi.org:10.1371/journal.ppat.1003341

6 Mentzer, A. J. *et al.* Identification of host-pathogen-disease relationships using a scalable multiplex serology platform in UK Biobank. *Nat Commun* **13**, 1818 (2022). https://doi.org:10.1038/s41467-022-29307-3

7 Cohen, J. I., Fauci, A. S., Varmus, H. & Nabel, G. J. Epstein-Barr virus: an important vaccine target for cancer prevention. *Sci Transl Med* **3**, 107fs107 (2011). https://doi.org:10.1126/scitranslmed.3002878

8 SoRelle, E. D. *et al.* Single-cell RNA-seq reveals transcriptomic heterogeneity mediated by host-pathogen dynamics in lymphoblastoid cell lines. *Elife* **10** (2021). https://doi.org:10.7554/eLife.62586

9 Chan, S. Y. *et al.* Authentication of nasopharyngeal carcinoma tumor lines. *Int J Cancer* **122**, 2169-2171 (2008). https://doi.org:10.1002/ijc.23374

10 Schmitz, R. *et al.* Burkitt lymphoma pathogenesis and therapeutic targets from structural and functional genomics. *Nature* **490**, 116-120 (2012). https://doi.org:10.1038/nature11378

11 Chen, Y. P. *et al.* Single-cell transcriptomics reveals regulators underlying immune cell diversity and immune subtypes associated with prognosis in nasopharyngeal carcinoma. *Cell Res* **30**, 1024-1042 (2020). https://doi.org:10.1038/s41422-020-0374-x

12 Gong, L. *et al.* Comprehensive single-cell sequencing reveals the stromal dynamics and tumor-specific characteristics in the microenvironment of nasopharyngeal carcinoma. *Nat Commun* **12**, 1540 (2021). https://doi.org:10.1038/s41467-021-21795-z

13 Jin, S. *et al.* Single-cell transcriptomic analysis defines the interplay between tumor cells, viral infection, and the microenvironment in nasopharyngeal carcinoma. *Cell Res* **30**, 950-965 (2020). https://doi.org:10.1038/s41422-020-00402-8

14 Liu, Y. *et al.* Tumour heterogeneity and intercellular networks of nasopharyngeal carcinoma at single cell resolution. *Nat Commun* **12**, 741 (2021). https://doi.org:10.1038/s41467-021-21043-4

15 Tang, K. W. & Larsson, E. Tumour virology in the era of high-throughput genomics. *Philos Trans R Soc Lond B Biol Sci* **372** (2017). https://doi.org:10.1098/rstb.2016.0265

16 Zhao, J. *et al.* Single cell RNA-seq reveals the landscape of tumor and infiltrating immune cells in nasopharyngeal carcinoma. *Cancer Lett* **477**, 131-143 (2020). https://doi.org:10.1016/j.canlet.2020.02.010

17 Tang, K. W., Alaei-Mahabadi, B., Samuelsson, T., Lindh, M. & Larsson, E. The landscape of viral expression and host gene fusion and adaptation in human cancer. *Nat Commun* **4**, 2513 (2013). https://doi.org:10.1038/ncomms3513

18 Borozan, I., Zapatka, M., Frappier, L. & Ferretti, V. Analysis of Epstein-Barr Virus Genomes and Expression Profiles in Gastric Adenocarcinoma. *J Virol* **92** (2018). https://doi.org:10.1128/JVI.01239-17

19 Griffin, B. E. & Karran, L. Immortalization of monkey epithelial cells by specific fragments of Epstein-Barr virus DNA. *Nature* **309**, 78-82 (1984). https://doi.org:10.1038/309078a0

20 Karran, L. *et al.* Establishment of immortalized primate epithelial cells with sub-genomic EBV DNA. *Int J Cancer* **45**, 763-772 (1990). https://doi.org:10.1002/ijc.2910450432

21 Arvey, A. *et al.* An atlas of the Epstein-Barr virus transcriptome and epigenome reveals host-virus regulatory interactions. *Cell Host Microbe* **12**, 233-245 (2012). https://doi.org:10.1016/j.chom.2012.06.008

22 Cancer Genome Atlas Research, N. Comprehensive molecular characterization of gastric adenocarcinoma. *Nature* **513**, 202-209 (2014). https://doi.org:10.1038/nature13480

23 Kaymaz, Y. *et al.* Comprehensive Transcriptome and Mutational Profiling of Endemic Burkitt Lymphoma Reveals EBV Type-Specific Differences. *Mol Cancer Res* **15**, 563-576 (2017). https://doi.org:10.1158/1541-7786.MCR-16-0305

24 Zhang, L. *et al.* Genomic Analysis of Nasopharyngeal Carcinoma Reveals TME-Based Subtypes. *Mol Cancer Res* **15**, 1722-1732 (2017). https://doi.org:10.1158/1541-7786.MCR-17-0134

25 Arvey, A. *et al.* The tumor virus landscape of AIDS-related lymphomas. *Blood* **125**, e14-22 (2015). https://doi.org:10.1182/blood-2014-11-599951

26 Holmqvist, I., Backerholm, A., Tian, Y., Xie, G., Thorell, K. & Tang, K. W. FLAME: long-read bioinformatics tool for comprehensive spliceome characterization. *RNA* **27**, 1127-1139 (2021). https://doi.org:10.1261/rna.078800.121

27 Chen, B., Khodadoust, M. S., Liu, C. L., Newman, A. M. & Alizadeh, A. A. Profiling Tumor Infiltrating Immune Cells with CIBERSORT. *Methods Mol Biol* **1711**, 243-259 (2018). https://doi.org:10.1007/978-1-4939-7493-1_12

28 Li, T. *et al.* Anti-Epstein-Barr Virus BNLF2b for Mass Screening for Nasopharyngeal Cancer. *N Engl J Med* **389**, 808-819 (2023). https://doi.org:10.1056/NEJMoa2301496

29 Jochum, S., Moosmann, A., Lang, S., Hammerschmidt, W. & Zeidler, R. The EBV immunoevasins vIL-10 and BNLF2a protect newly infected B cells from immune recognition and elimination. *PLoS Pathog* **8**, e1002704 (2012). https://doi.org:10.1371/journal.ppat.1002704

30 Bruce, J. P. *et al.* Whole-genome profiling of nasopharyngeal carcinoma reveals viral-host co-operation in inflammatory NF-kappaB activation and immune escape. *Nat Commun* **12**, 4193 (2021). https://doi.org:10.1038/s41467-021-24348-6

31 Love, M. I., Huber, W. & Anders, S. Moderated estimation of fold change and dispersion for RNA-seq data with DESeq2. *Genome Biol* **15**, 550 (2014). https://doi.org:10.1186/s13059-014-0550-8

32 Yamawaki, T. M. *et al.* Systematic comparison of high-throughput single-cell RNA-seq methods for immune cell profiling. *BMC Genomics* **22**, 66 (2021). https://doi.org:10.1186/s12864-020-07358-4

33 Ziegler, P. *et al.* A primary nasopharyngeal three-dimensional air-liquid interface cell culture model of the pseudostratified epithelium reveals differential donor- and cell type-specific susceptibility to Epstein-Barr virus infection. *PLoS Pathog* **17**, e1009041 (2021). https://doi.org:10.1371/journal.ppat.1009041

34 Holmgren, L. *et al.* Horizontal transfer of DNA by the uptake of apoptotic bodies. *Blood* **93**, 3956-3963 (1999).

35 Subramanian, A. *et al.* Gene set enrichment analysis: a knowledge-based approach for interpreting genome-wide expression profiles. *Proc Natl Acad Sci U S A* **102**, 15545-15550 (2005). https://doi.org:10.1073/pnas.0506580102

36 Tay, J. K. *et al.* The microdissected gene expression landscape of nasopharyngeal cancer reveals vulnerabilities in FGF and noncanonical NF-kappaB signaling. *Sci Adv* **8**, eabh2445 (2022). https://doi.org:10.1126/sciadv.abh2445

37 Ungerleider, N. *et al.* EBV miRNAs are potent effectors of tumor cell transcriptome remodeling in promoting immune escape. *PLoS Pathog* **17**, e1009217 (2021). https://doi.org:10.1371/journal.ppat.1009217

38 Gaude, E. & Frezza, C. Tissue-specific and convergent metabolic transformation of cancer correlates with metastatic potential and patient survival. *Nat Commun* **7**, 13041 (2016). https://doi.org:10.1038/ncomms13041
39 Albanese, M. *et al.* Epstein-Barr virus microRNAs reduce immune surveillance by virus-specific CD8+ T cells. *Proc Natl Acad Sci U S A* **113**, E6467-E6475 (2016). https://doi.org:10.1073/pnas.1605884113
40 Jiang, P. *et al.* Systematic investigation of cytokine signaling activity at the tissue and single-cell levels. *Nat Methods* **18**, 1181-1191 (2021). https://doi.org:10.1038/s41592-021-01274-5
41 Riley, K. J., Rabinowitz, G. S., Yario, T. A., Luna, J. M., Darnell, R. B. & Steitz, J. A. EBV and human microRNAs co-target oncogenic and apoptotic viral and human genes during latency. *EMBO J* **31**, 2207-2221 (2012). https://doi.org:10.1038/emboj.2012.63
42 Yu, H. & Jove, R. The STATs of cancer--new molecular targets come of age. *Nat Rev Cancer* **4**, 97-105 (2004). https://doi.org:10.1038/nrc1275
43 Sakiragaoglu, O. & Munn, A. L. Inhibition of Telomerase Activity Using an EGFP-Intron Splicing System Encoding Multiple RNAi Sequences. *Mol Biotechnol* **58**, 832-837 (2016). https://doi.org:10.1007/s12033-016-9982-6
44 Androvic, P., Valihrach, L., Elling, J., Sjoback, R. & Kubista, M. Two-tailed RT-qPCR: a novel method for highly accurate miRNA quantification. *Nucleic Acids Res* **45**, e144 (2017). https://doi.org:10.1093/nar/gkx588
45 Hao, Y. *et al.* Integrated analysis of multimodal single-cell data. *Cell* **184**, 3573-3587 e3529 (2021). https://doi.org:10.1016/j.cell.2021.04.048
46 Robin, X. *et al.* pROC: an open-source package for R and S+ to analyze and compare ROC curves. *BMC Bioinformatics* **12**, 77 (2011). https://doi.org:10.1186/1471-2105-12-77

### Figure Legends

**Figure 1. Detection and characterization of EBV gene expression**

**a**, RNA-Seq data from four types of neoplasms and two types of EBV cell lines were analyzed for their EBV-content. The viral RNA from datasets containing more than 10 ppm EBV RNA were plotted against the EBV genome. Further sub-analyses were conducted on the EBV-positive tumors (box). **b**, Fraction of datasets with high (>10 ppm, red) or low (2-10 ppm, grey) EBV content. Numbers in parentheses indicate the number of patients for each category and percentages represent the fraction of datasets with high EBV content. **c**, Average coverage of EBV RNA in the four types of neoplasms. Numbers on the x-axis correspond to the EBV genome position. Alignment to the BamHI digestion map is shown in the bottom. **d**, Magnification of the RNA reads within the BamHI-A/I region. Rightward *RPMS1* exons (in roman numerals) and leftward genes depicted in the bottom. Unique regions without overlap with other genes are shown in dotted boxes. **e**, RNA coverage of transcription start site of *RPMS1* in NPC1. (F) Splice-junction reads detected within the BamHI-A/I region in NPC1. Lines between the constitutive exons of *RPMS1* are shown in bold. **g**, poly-A containing reads detected at the 3'-end of *RPMS1* in NPC1. Reference sequence and poly-A signal shown in the bottom. ppm, parts per million reads; NPC, nasopharyngeal carcinoma; GAC, gastric adenocarcinoma; eBL, endemic Burkitt's lymphoma; sBL, sporadic Burkitt's lymphoma; ECL, EBV associated tumor derived cell line; LCL, lymphoblastic cell line.

**Figure 2. EBV gene expression in bulk RNA sequencing data**

**a-f**, Heatmap depicting tpm-values of four gene regions *RPMS1/BAREs*, *LMP1/BNLF2*, *LMP2A/B* and *EBNA1* in NPC, GAC, eBL, sBL, ECL and LCL. Three datasets containing additional EBV genes expressed at more than 5 tpm are marked with an asterisk. These datasets contained EBV genes indicative of lytic replication within the neoplasm. **g-i**, tpm-distribution of genes with overlapping 3'. The fused RPMS1/BAREs 3' end, compared with the unique regions for RPMS1, BARE1, BARE2 and BARE3, the fused LMP1/BNLF2 3' end, compared with the unique regions for LMP1 and the fused LMP2A/2B 3' end, compared with the unique regions for LMP2A, LMP2B and BNRF1 in NPC, GAC, eBL and sBL. **j**, Generalized pathway perturbations in principal components for respective cancer type. Principal components correlating with highest EBV-status separation are marked with bold. tpm, transcripts per million reads; NPC, nasopharyngeal carcinoma; GAC, gastric adenocarcinoma; eBL, endemic Burkitt's lymphoma; sBL, sporadic Burkitt's lymphoma; PC, principal component.

**Figure 3. Nasopharyngeal carcinoma single-cell RNA sequencing datasets**

**a**, Proportion of epithelial and stromal cells in the four studies. Striped portion of the bar shows the fraction of EBV-expressing cells in each category. **b**, Classification of each sample according to their origin and EBV-status according to EBER in situ hybridization, antibody detection or UMI in the single-cell data. Samples showing concordant results from two analyses are shown in green, discordant in purple and unknown in grey. **c**, EBV expression in datasets containing more than 100 epithelial cells (green). The proportion of epithelial cells from each tumor expressing fused EBV gene is shown in the respective column (orange). Genes expressed over 2 cpm were included. **d**, Inferred chromosomal RNA expression throughout the genome in T-cells (upper panel) and epithelial cells (lower panel), position on x-axis correspond to position in respective chromosome. Epithelial cells divided by unsupervised hierarchical clustering. Areas in red depicts inferred gains and blue loss of chromosomal segment. **e-g**, Epithelial cells extracted from NPC1 were reclustered in UMAP. Cancer cells classified according to EBV expression (blue) showed a lower sensitivity compared to cancer (red) and healthy cell classification based on inference of chromosomal copy number variation. NPC, nasopharyngeal carcinoma; EBER, Epstein–Barr virus–encoded small RNAs; UMI, unique molecular identifier; cpm, counts per million reads; UMAP, uniform manifold approximation and projection.

**Figure 4. EBV-induced host perturbations**

**a**, Changes in biological pathways between cancer cells and healthy cells from the same patients. Hallmarks enriched in all four EBV-positive NPCs studies are listed. The same pathways for EBV-negative tumors and healthy controls in which basal cells were compared to differentiated cells in non-tumor samples are shown alongside. Absence of bar indicates no significant differences. Induced changes in the EBV-negative nasopharyngeal carcinoma cell line HK1 transfected with *RPMS1* miR-BARTs (circles) and Namalwa cells treated with doxycycline (triangles) to upregulate *RPMS1* gene are shown in the right column. **b**, Genes perturbed in the same direction in multiple tumors. Enriched genes are marked with green (upregulated) and magenta (downregulated). The x-axis shows the negative log10 of the false discovery rate q-value (FDRq) for pathways in respective category. **c**, Immune response genes downregulated in tumor cells categorized according to pathway. Genes in italics are also part of the NF-κB pathway. **d**, Depiction of factors involved in viral perturbations in NPC epithelial cells. MITSP, mitotic spindle; UVD, UV responsed down; IFNA, interferon alpha response; IFNG, interferon gamma response; OXPH, oxidative phosphorylation; IFN, interferon.

### Methods

#### Patient datasets

The transcriptome datasets of the primary tumor tissues and the cell lines were downloaded from several databases. List of the datasets can be found in Extended Data Table 1.

#### Bulk RNA analyses

The raw reads were quality filtered using PRINSEQ/0.20.3. The sequencing adapters were removed using TrimGalore/0.4.4. The reads were aligned towards human (Grch38) and EBV (NC_007605.1) respectively with STAR/2.5.2b. Both alignment files were filtered to allow 10 multimapped reads, 3 mismatches and a minimum alignment length of 40 nucleotides. Due to the limitations of the sequencing datasets where short RNAs were not included into the sequencing library the remaining reads mapping to EBV encoded RNAs (EBERs) have been artificially omitted. Additional information regarding analysis of *RPMS1* transcription start site, BamHI-A/I splice-junctions and EBV gene expression can be found in Supplementary Information. Polyadenylation signal analysis was conducted by identifying all reads containing the termination polyA-signal, and polyA-stretches found 10-30 basepairs downstream of the polyA-signal (Extended Data Table 2).

#### Single-cell RNA analyses

The NPC single cell RNA datasets was mapped using Cellranger/3.0.2 against the modified Akata EBV reference genome and the human reference genome GRCh38 (hg38, UCSC)[33]. Cells with less than 200 or more than 9000 genes were removed from the cellranger filtered matrix and all the counts in remaining cells were normalized using the R package sctransform default settings. The principal component analysis of all genes in remaining cells was performed to compute 100 principal components and the first 20 were used for the additional dimensionality reduction and visualization of the cells using UMAP (k=30).

The cell type of different clusters were annotated based on the distribution and expression of canonical marker gene sets or SingleR according to the human cell atlas. Each sample was analyzed separately in order to avoid the batch effects.The epithelial-like cluster which was not annotated as epithelial by singleR but expressed a high level of epithelial cell markers were classified as epithelial cells manually.

The total unique molecular identifier (UMI) count of every EBV gene in each sample was counted and used for the calculations. EBV counts per million reads (cpm) is the EBV total UMI in each cell/sample divided by the total UMI count of both viral and host genes in that cell/sample. By extracting the EBV gene UMI in each epithelial cell and the total UMI per cell, the single-cells as bulk heatmaps were created. The UMI-features (genes) matrix of the epithelial cells from each sample was extracted using Seurat, followed by re-normalization and sub-clustering the epithelial cells. Due to the variation of the number of epithelial cells in the samples, only selected datasets were included in this analysis. The criteria for inclusion are 1) the sample contained epithelial cells, including both healthy and tumor; 2) the sample had at least two sub-epithelial cell clusters; 3) both EBV positive and negative cell clusters could be separated. The malignant and healthy cells from each selected sample were then utilized for comparison. The number of differentially expressed genes without filtering were added into Supplementary Table 7. The genesets selected based on cutoffs of log2 fold change and p-adjusted values were utilized for the enriched hallmarks profiling by GSEA.

**Cells, plasmids and chemicals**

The nasopharyngeal carcinoma cell lines C666-1 and HK1, and the Burkitt's lymphoma cell line Namalwa were grown in RPMI-1640 medium (Gibco) supplemented with 10% fetal calf serum and cultured at 37°C with 5% $CO_2$.

To generate a Tet-On 3G-Expressing stable Namalwa cell line, 1ug of pCMV-Tet3G plasmid was transfected in $5.6x10^5$ Namalwa cells by electroporation (Thermo Neon Transfection System). Positive cells were selected by G418 (800ug/ml) over two weeks. Plasmids pTRE3G-BI-mCherry, pCMV-Tet3G and linear selection Marker (puromycin) were purchased from Takara. The CRISPR/Cas9 plasmid (px458) was purchased from Addgene. To construct the template for Cas9-triggered homologous recombination, a fragment containing homology arms (NC_007605, 5':137469-138267 and 3':138335-138946) and the mCherry with bidirectional promoter were cloned into a pUC19 vector by using DNA assembly cloning kit (NEB, E5520S). The fragment used for replacing the endogenous *RPMS1* promoter containing homology arms at both ends, mCherry and the bidirectional promoter were amplified by PCR and purified by PCR Cleanup Kit (NEB). The *RPMS1* promoter was recognized by dual sgRNAs inserted within px458, targeting 138265-138284 and 138332-138351 respectively. Five days post-transfection, cells were sorted as single cells into 96-well plates and cultured for six weeks. The *RPMS1* promoter replaced by an inducible bidirectional promoter encoding mCherry, between the Cas9 target sites was confirmed by whole genome sequencing. The plasmid used for overexpressing *RPMS1* long non-coding RNA was 17ADVGAP, the vector with the entire long non-coding *RPMS1* cDNA, which was ordered from GeneArt. The sequence encoding miR-BARTs clusters was cloned from C666-1 and inserted into the blue fluorescent protein gene as an intron[43].

RPMS1-FISH was performed using the ViewRNA Cell Plus Assay (#88–1900, Affymetrix) according to the manufacturer's protocol. After fixation and permeabilization, Cell Plus Probe Solution was prepared by diluting Probe Sets 1:100 in pre-warmed Cell Plus Probe Set Diluent and vortexing briefly to mix. The cells were overlaid with Cell Plus Probe Solution (400 µl per well) and gently rocked to mix and distribute the diluted target probe for 2 h at 40 ± 1 °C in a validated incubator. Next, we aspirated the Cell Plus Probe Solution and gently and extensively washed the cells with the Cell Plus RNA Wash Buffer Solution using a dropper or pipette to slowly and carefully add 800 µl per well. The cells were covered with Wash Buffer Solution for 24 h at 4 °C in the dark. The next day, the samples were pre-warmed to room temperature. The Cell Plus RNA Wash Buffer Solution was aspirated, and the cells were overlaid with Cell Plus Amplifier Diluent (400 µl with 15 µl Cell Plus PreAmplifier Mix) for 1 h at 40 ± 1 °C in a validated incubator. The cells were washed extensively, counterstained with DAPI on the slides and mounted with Antifade Reagent (#p36930, Invitrogen). The *RPMS1* probe set was designed by custom service and ordered from AH Diagnostics.

Working solutions of 3 mM sodium butyrate (Alfa Aesar) and 32 nM (20 ng/ml) phorbol 12-myristate 13-acetate/12-O-tetradecanoylphorbol-13-acetate (Fisher Bioreagents) were made in distilled water and DMSO, respectively. C666-1 cells were seeded at an initial concentration of 4 x $10^5$ cells/mL. 24 hours after subculture, cells were incubated in fresh medium supplemented with chemical inducing agents. Total RNA was extracted at indicated time points using TRIzol reagent (Life Technologies).

### Sequencing

Nanopore single-molecule long-read sequencing was performed as previously described[26]. In brief, total RNA was extracted from C666-1 using TRIzol and subsequently treated with TURBO DNA-free Kit (Thermo Fisher). Libraries were prepared using a PCR-cDNA approach with forward primers at variable positions and a common barcoded reverse primer (Supplementary Table 8). Total RNA (1.5 to 2 μg) was reverse transcribed followed by 40 cycles of PCR amplification. Pooled libraries were sequenced on a MinION Mk1B device (MIN-101B) and fast5 files were basecalled using Guppy (v3.6.1+249406c, dna_r9.4.1_450bps_hac, default settings). Minimap2 was used for splice-aware alignment to the EBV genome. Long-read splicing analysis was performed using FLAME.

Promoter replaced (ProRe) Namalwa cells was submitted to Dante Labs for whole genome sequencing. In total, 974,808,218 and 47,082 sequencing reads were mapped to the human reference genome and EBV reference genome, respectively. This resulted in a sequencing depth of coverage of 45.69x and 41.10x for the human sequences and the EBV sequences, respectively.

Total RNA from the Namalwa cell lines and C666-1 cell line was extracted using TRIzol reagent (Life Technologies) according to the supplier's instructions. RNA yield was determined spectrophotometrically by measuring the absorbance at 260 nm (NanoDrop 2000). The eluate was subjected to DNase treatment (TURBO DNA-free™ Kit, Thermo Fisher Scientific) and then stored at -80°C. Stranded cDNA libraries preparation and paired-end sequencing were performed at GENEWIZ (Germany). The sequencing data was processed as mentioned above. The Namalwa derived cell lines were sequenced in triplicates, in total 21 datasets in this study.

MicroRNA from the ProRe Namalwa cell line was extracted using the MiRNeasy Serum/Plasma Advanced Kit (Qiagen) after 48h doxycycline treatment. The quantity of the RNA was measured by Qubit. A microRNA library was prepared using QIAseq miRNA library kit and sequenced by IIlumina MiniSeq System with High-Output Kit.

**Quantitative PCR**

Control *RPMS1* RNA was generated by MEGAscript T7 Transcription Kit (Thermo Fisher) according to the manufacturer's protocol. After purification, the RNA concentration was measured by Nanodrop. $8.87x10^6$ copies of control *RPMS1* RNA were added to the TRIzol lysis of $1.49x10^6$ Namalwa cells as a spike-in control for RNA extraction and RT-qPCR. RT-qPCR was performed using SuperScript III Platinum One-Step RT-qPCR Kit (Thermo). The Ct values of control (without spike-in) and experiment (with spike-in) were used to calculate the copy number of endogenous *RPMS1* transcripts.

The expression of *BZLF1* and *RPMS1* was assessed by RT-qPCR after DNA removal using TURBO DNA-free™ Kit (Life Technologies). cDNA synthesis was performed using High-Capacity cDNA Reverse Transcription Kit (Thermo Fisher) according to the supplier's instructions. The reverse transcription reaction mixture was subsequently diluted 1:3 and a 1.5 µl-portion was used for qPCR. Each qPCR reaction was assembled in a total volume of 20 µl and contained 2x TATAA SYBR GrandMaster Mix (TATAA Biocenter) and 0.5 µM of each primer. The following cycling conditions were used: 95°C for 3 min followed by 45 cycles with 95°C for 10, 60°C for 30 s and 72°C for 30 s. Comparative quantification of gene expression was done using the ΔΔCt method with B-actin as normalizer and an untreated sample at each time point as calibrator. Results were analyzed from three technical replicates.

HK1 cells were transfected 24 hours after passaging into a 24-well plate at 70% confluence with Lipofectamine 3000 according to the manufacturer's instructions (Invitrogen). Expression plasmids used for transfections included C1-mCherry (Control) and C1-BFP-miR-BARTs. After 48 hours of transfection, total RNA was extracted by TRIzol and treated with TURBO

DNA-free Kit. Two-tailed RT-qPCR was performed according to the manufacturer's instructions[44]. The two-tailed RT and qPCR primers for miR-BARTs were designed by TATAA Biocenter.

### Statistical analysis

All statistical analyses were performed using R packages Seurat[45], ggpubr, and DESeq2[31]. Pathway enrichment analysis was done using GSEA[35]. ROC-AUC was performed using the R package pROC[46] while PCA on the bulk RNA-seq data was performed using the R package stats.

### Data availability

Source data are provided upon publication.

### Acknowledgements

We thank Dr. George Tsao, University of Hong Kong, for the generous gift of the C666-1 and HK1 cell line. The results shown here are in part based upon data generated by the TCGA Research Network: https://www.cancer.gov/tcga. The computations and data handling were enabled by resources provided by the Swedish National Infrastructure for Computing (SNIC, project sens2018120) at Uppsala Multidisciplinary Center for Advanced Computational Science (UPPMAX) partially funded by the Swedish Research Council through grant agreement no. 2018-05973. We thank the Bioinformatics Core Facility and the Centre for Cellular Imaging at the Sahlgrenska Academy for bioinformatics and miscroscopy analyses respectively. KHYS was supported by National Institute of Allergy and Infectious Diseases (R01AI168011). This study was supported by grants from Svenska Sällskapet för Medicinsk Forskning (S21-0083), Vetenskapsrådet (2023-02292), Assar Gabrielssons Research Foundation, Region Västra Götaland, Sweden.

### Author contributions

K-W.T. and Y.T. conceived the study. Y.H., K.H.Y.S. and A.B. designed and implemented epithelial cell classification. Y.T., G.X., A.B., I.H., D.V., J.L. S.A. and J.C. collected and analyzed the data under the supervision of Y.H., K.H.Y.S. and K-W.T.. K-W.T., Y.T., G.X. and A.B. prepared the manuscript. All authors reviewed and edited the manuscript.

### Competing interest declaration

All authors declare no competing interests.

### Additional Information

### Extended data figure and tables legends

**Extended Data Figure 1. EBV RNA tumors and cell lines**

**a**, The fraction of EBV-content in each dataset was quantified. Samples containing more than 10 ppm EBV RNA were considered positive. EBV ppm-distribution in EBV-positive neoplasms and cell lines. **b**, Fraction of EBV reads aligning to the BamHI-A/I region in neoplasms and cell lines. The majority of EBV reads in primary samples originated from a single region, the BamHI-A/I region, of the EBV genome. NPC, nasopharyngeal carcinoma; GAC, gastric adenocarcinoma; eBL, endemic Burkitt's lymphoma; sBL, sporadic Burkitt's lymphoma; ECL, EBV-associated tumor derived cell lines; LCL, lymphoblastoid cell lines.

**Extended Data Figure 2. Peak analysis of RNA in the BamHI-A/I region**

**a**, Heatmap of peaks above 15% of top value in each tumor within the BamHI-A/I region. Each row represents a single tumor and each column a genome segment of the region. Red depicts areas where peaks were detected. **b**, Percentage of datasets with peaks in the segments for each cancer type. Alignment to genes encoded within the region (bottom), the exons of *RPMS1* are marked with roman numerals. **c**, Novel BARE transcript variants. C666-1 RNA coverage of *RPMS1/BAREs* from short-read sequencing data. The rightward *RPMS1* exons are depicted as black boxes/arrow and leftward genes are shown in blue. **d**, Magnification of region with novel BamHI-A rightward elements (*BAREs*). Single-molecule long-read sequencing of non-overlapping regions with starting positions of the three new genes *BARE1* (red), *BARE2* (green) and *BARE3* (turquoise). **e**, The two most common transcript variants of each *BARE* aligned to the BamHI-A region. Splicing for *BARE1* is observed at genome position 153,528.

**Extended Data Figure 3. Calculation of *RPMS1* copy number in Namalwa cells**

**a**, Confocal microscopy of *RPMS1* RNA *in situ* hybridization showed a variation in the number of foci in the nucleus. Cell nuclei were counterstained with DAPI. Multiple focal planes of a single region (red square) with positive and negative cells. **b**, Quantitative PCR of *RPMS1* showed an average value of two RNA copies per cell. **c**, Expression levels (tpm-values) of housekeeping genes for all EBV-positive neoplasms. Datasets were sorted in a decreasing order according to the EBV ppm levels within each tumor type. NPC, nasopharyngeal carcinoma; GAC, gastric adenocarcinoma; eBL, endemic Burkitt's lymphoma; sBL, sporadic Burkitt's lymphoma. tpm, transcripts per million

**Extended Data Figure 4. Normalized EBV gene expression in neoplasms and cell lines**

**a-f**, Tpm-values of EBV genes in NPC, GAC, eBL, sBL, ECL and LCL. Datasets from primary tumors with global EBV gene expression indicative of EBV replication are marked in colour (NPC3, green; eBL1, blue; eBL5, red). Tpm, transcripts per million; NPC, nasopharyngeal carcinoma; GAC, gastric adenocarcinoma; eBL, endemic Burkitt's lymphoma; sBL, sporadic Burkitt's lymphoma.

**Extended Data Figure 5. Gene set enrichment assay of bulk sequencing datasets**

**a**, Pathway perturbations for each principal component for all tumor types. **b-e**, Calculation of Euclidean distance between EBV-positive and EBV-negative samples for each tumor type.

**Extended Data Figure 6. Nasopharyngeal carcinoma single-cell RNA sequencing datasets**

**a**, Cell type characterization for each sample. **b**, Fraction of EBV-expressing cells in each cell type. Cell types with fewer than 10 cells were omitted. Epi, epithelial cell; B, B lymphocyte; T, T lymphocyte; NK, natural killer cell; Mye, myeloid cell; Others, other cell types.

**Extended Data Figure 7. Merged EBV gene expression**

The tumors were separated according to the library preparation chemistry used for each study. **a-c**, The average cpm-value of EBV genes of entire NPC single cell dataset analyzed as bulk. Tumors from Study 2 were omitted from this analysis due to epithelial cell enrichment. **d-f**, The average cpm-value of EBV genes in epithelial cells. cpm, counts per million reads.

**Extended Data Figure 8. Genes set enrichment analysis of cancer cells**

Cancer cells were compared to their healthy counterparts in each tumor. Variance of gene expression was analyzed by genes set enrichment analysis and significant perturbations were plotted for each study to compensate for batch effect. Tumor cells from EBV-negative samples (EBV-) were compared to healthy cells from the same tissue sample, and basal cell were compared to differentiated cells in non-tumor samples (Controls). Absence of bar indicates no significant differences. Induced changes in the EBV-negative nasopharyngeal carcinoma cell line HK1 transfected with *RPMS1* miR-BARTs (circles) and Namalwa cells treated with doxycycline (triangles) to upregulate *RPMS1* gene are shown in the right column (Cell lines).

**Extended Data Figure 9. EBV microRNA expression**

**a**, MicroRNA expression in BL, GAC and ProRe normalized according to falling expression levels in patient samples. **b**, Two-tailed PCR of EBV microRNA in HK1-cells transfected with plasmids encoding miR-BARTs in the introns. **c**, Down-regulation of interferon stimulated genes in HK1-cells transfected with plasmids encoding miR-BARTs. BL, Burkitt's lymphoma; GAC gastric adenocarcinoma; ProRe, Namalwa cells with replaced inducible promoter.

**Extended Data Figure 10. Expression of cytokines and IFN pathway**

**a**, Cytokines verified to be expressed in bulk sequencing NPC datasets were quantified in the three cell types (epithelial, B and T cells) found in almost all tumors. The size of the dot depicts the percentage of respective cell types which expressed specific cytokines. The intensity of the colour corresponds to the mean expression level of the cytokine in respective cell type compared with average expression in the other cell types. **b**, Interferon receptor and STAT-expression in cancer cells compared with healthy epithelial cells in EBV-positive and EBV-negative tumors as well as undifferentiated epithelial cells compared with differentiated epithelial cells in control biopsies, divided by study.

**Supplementary Table 1 - List of datasets**

**Supplementary Table 2 - PolyA-containing reads**

**Supplementary Table 3 - House-keeping genes tpm values**

**Supplementary Table 4 - EBV genes tpm values**

**Supplementary Table 5 - List of cell amount in the single-cell datasets**

**Supplementary Table 6 - Cpm values of EBV genes in scRNA-Seq as bulk**

**Supplementary Table 7 - Differential gene expression for NPC**

**Supplementary Table 8 - Primers and oligonucleotides**

## Figures

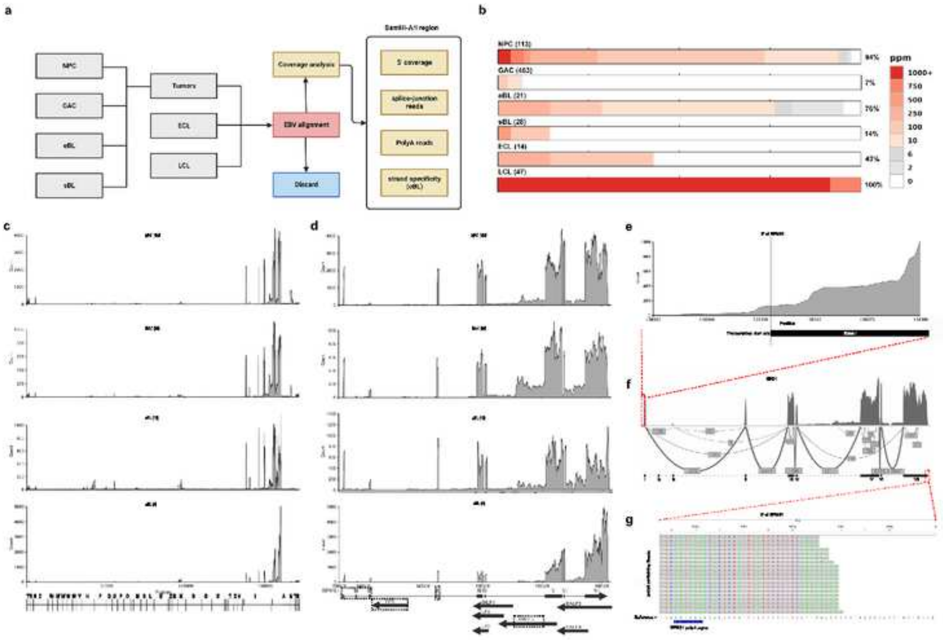

### Figure 1

Detection and characterization of EBV gene expression a, RNA-Seq data from four types of neoplasms and two types of EBV cell lines were analyzed for their EBV-content. The viral RNA from datasets containing more than 10 ppm EBV RNA were plotted against the EBV genome. Further sub-analyses were conducted on the EBV positive tumors (box). b, Fraction of datasets with high (>10 ppm, red) or low (2-10 ppm, grey) EBV content. Numbers in parentheses indicate the number of patients for each category and percentages represent the fraction of datasets with high EBV content. c, Average coverage of EBV RNA in the four types of neoplasms. Numbers on the x-axis correspond to the EBV genome position. Alignment to the BamHI digestion map is shown in the bottom. d, Magnification of the RNA reads within the BamHI-A/I region. Rightward RPMS1 exons (in roman numerals) and leftward genes depicted in the bottom. Unique regions without overlap with other genes are shown in dotted boxes. e, RNA coverage of transcription start site of RPMS1 in NPC1. (F) Splice-junction reads detected within the BamHI-A/I region in NPC1. Lines between the constitutive exons of RPMS1 are shown in bold. g, poly-A containing reads detected at the 3'-end of RPMS1 in NPC1. Reference sequence and poly-A signal shown in the bottom. ppm, parts per million reads; NPC, nasopharyngeal carcinoma; GAC, gastric adenocarcinoma; eBL, endemic Burkitt's lymphoma; sBL, sporadic Burkitt's lymphoma; ECL, EBV associated tumor derived cell line; LCL, lymphoblastic cell line.

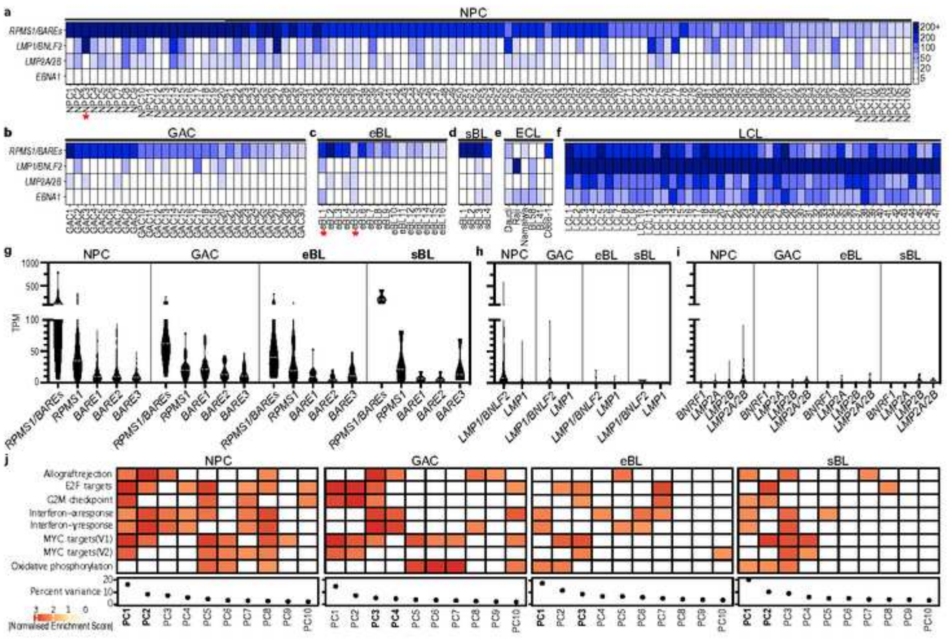

### Figure 2

EBV gene expression in bulk RNA sequencing data a-f, Heatmap depicting tpm-values of four gene regions RPMS1/BAREs, LMP1/BNLF2, LMP2A/B and EBNA1 in NPC, GAC, eBL, sBL, ECL and LCL. Three datasets containing additional EBV genes expressed at more than 5 tpm are marked with an asterisk. These datasets contained EBV genes indicative of lytic replication within the neoplasm. g-i, tpm-distribution of genes with overlapping 3'. The fused RPMS1/BAREs 3' end, compared with the unique regions for RPMS1, BARE1, BARE2 and BARE3, the fused LMP1/BNLF2 3’ end, compared with the unique regions for LMP1 and the fused LMP2A/2B 3’ end, compared with the unique regions for LMP2A, LMP2B and BNRF1 in NPC, GAC, eBL and sBL. j, Generalized pathway perturbations in principal components for respective cancer type. Principal components correlating with highest EBV-status separation are marked with bold. tpm, transcripts per million reads; NPC, nasopharyngeal carcinoma; GAC, gastric adenocarcinoma; eBL, endemic Burkitt’s lymphoma; sBL, sporadic Burkitt’s lymphoma; PC, principal component.

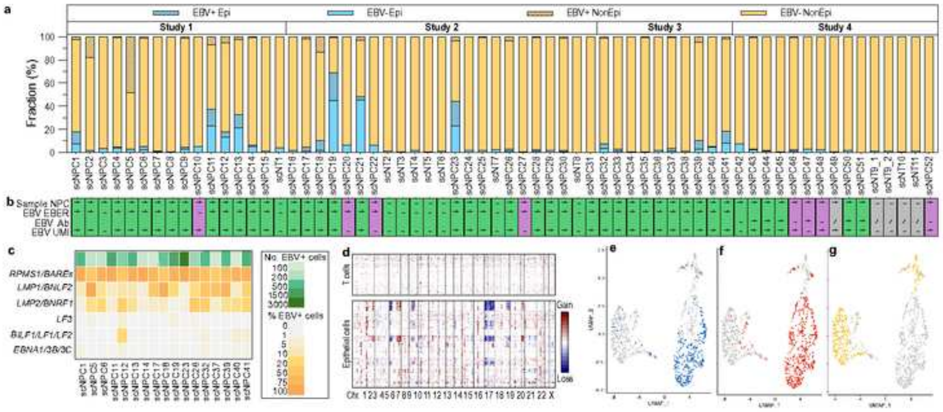

### Figure 3

Nasopharyngeal carcinoma single-cell RNA sequencing datasets a, Proportion of epithelial and stromal cells in the four studies. Striped portion of the bar shows the fraction of EBV-expressing cells in each category. b, Classification of each sample according to their origin and EBV-status according to EBER in situ hybridization, antibody detection or UMI in the single-cell data. Samples showing concordant results from two analyses are shown in green, discordant in purple and unknown in grey. c, EBV expression in datasets containing more than 100 epithelial cells (green). The proportion of epithelial cells from each tumor expressing fused EBV gene is shown in the respective column (orange). Genes expressed over 2 cpm were included. d, Inferred chromosomal RNA expression throughout the genome in T-cells (upper panel) and epithelial cells (lower panel), position on x-axis correspond to position in respective chromosome. Epithelial cells divided by unsupervised hierarchical clustering. Areas in red depicts inferred gains and blue loss of chromosomal segment. e-g, Epithelial cells extracted from NPC1 were reclustered in UMAP. Cancer cells classified according to EBV expression (blue) showed a lower sensitivity compared to cancer (red) and healthy cell classification based on inference of chromosomal copy number variation. NPC, nasopharyngeal carcinoma; EBER, Epstein–Barr virus–encoded small RNAs; UMI, unique molecular identifier; cpm, counts per million reads; UMAP, uniform manifold approximation and projection.

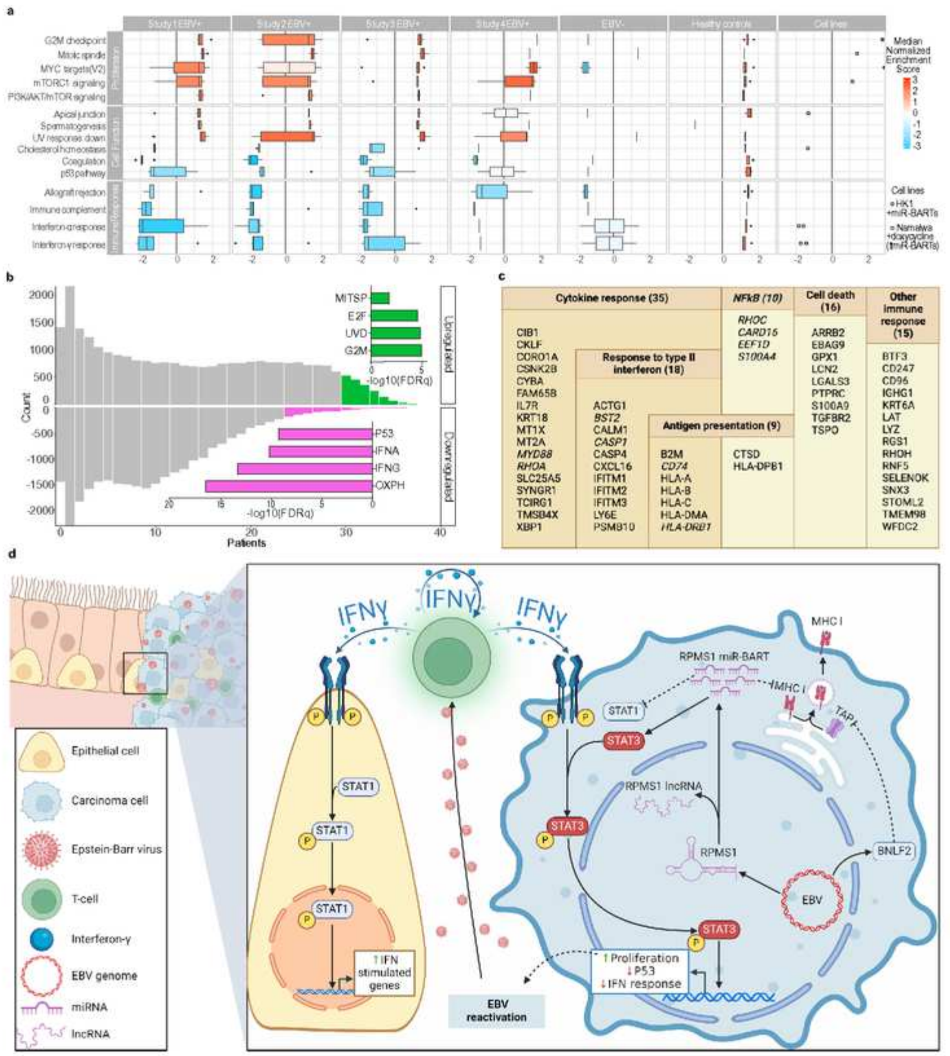

### Figure 4

EBV-induced host perturbations a, Changes in biological pathways between cancer cells and healthy cells from the same patients. Hallmarks enriched in all four EBV-positive NPCs studies are listed. The same pathways for EBV-negative tumors and healthy controls in which basal cells were compared to differentiated cells in non-tumor samples are shown alongside. Absence of bar indicates no significant differences. Induced changes in the EBV-negative nasopharyngeal carcinoma cell line HK1 transfected with RPMS1 miR-BARTs (circles) and Namalwa cells treated with doxycycline (triangles) to upregulate RPMS1 gene are shown in the right column. b, Genes perturbed in the same direction in multiple tumors. Enriched genes are marked with green (upregulated) and magenta (downregulated). The x-axis shows the negative log10 of the false discovery rate q-value (FDRq) for pathways in respective category. c, Immune response genes downregulated in tumor cells categorized according to pathway. Genes in italics are also part of the NF-κB pathway. d, Depiction of factors involved in viral perturbations in NPC epithelial cells. MITSP, mitotic spindle; UVD, UV responsed down; IFNA, interferon alpha response; IFNG, interferon gamma response; OXPH, oxidative phosphorylation; IFN, interferon.

### Supplementary Files

This is a list of supplementary files associated with this preprint. Click to download.

- ExtendedDataTable1Listofdatasets.xlsx
- ExtendedDataTable2PolyAcontainingreads.xlsx
- ExtendedDataTable3Housekeepinggenestpmvalues.xlsx
- ExtendedDataTable4EBVgenestpmvalues.xlsx
- ExtendedDataTable5ListofcellnumbersinscRNASeq.xlsx
- ExtendedDataTable6CpmvaluesofEBVgenesinscRNASeqasbulk.xlsx
- ExtendedDataTable7DifferentialgeneexpressionforNPC.xlsx
- ExtendedDataTable8Primersandoligonucleotides.xlsx
- ExtendeddataFigure1EBVRNA.pdf
- ExtendeddataFigure2RNApeakanalysis.pdf
- ExtendeddataFigure3RPMS1copynumber.pdf
- ExtendedDataFigure4EBVexpression.pdf
- ExtendedDataFigure5BulkGSEA.pdf
- ExtendedDataFigure6Stromalcellcomposition.pdf
- ExtendedDataFigure7Singlecellexpressionmerged.pdf
- ExtendedDataFigure8SinglecellGSEA.pdf
- ExtendedDataFigure9EBVmicroRNAexpression.pdf
- ExtendedDataFigure10Expressionofcytokinesandifnpathway.pdf
- SupplementaryInformation.pdf
